# CyberKnife Stereotactic Radiosurgery for Recurrent, Metastatic, and Residual Hemangiopericytomas

**DOI:** 10.1186/1756-8722-4-26

**Published:** 2011-06-06

**Authors:** Anand Veeravagu, Bowen Jiang, Chirag G Patil, Marco Lee, Scott G Soltys, Iris C Gibbs, Steven D Chang

**Affiliations:** 1 Department of Neurosurgery, Stanford University School of Medicine, Stanford, CA, USA; 2 Department of Neurosurgery, Santa Clara Valley Medical Center, San Jose, CA, USA; 3 Department of Radiation Oncology, Stanford University School of Medicine, Stanford, CA, USA

## Abstract

**Objective:**

Hemangiopericytoma is a rare and aggressive meningeal tumor. Although surgical resection is the standard treatment, hemangiopericytomas often recur with high incidences of metastasis. The purpose of this study was to evaluate the role of CyberKnife stereotactic radiosurgery (CK) in the management of recurrent, metastatic, and residual hemangiopericytomas.

**Methods:**

In a review of the Stanford radiosurgery database between 2002 and 2009, the authors found 14 patients who underwent CK therapy for recurrent, metastatic, and residual hemangiopericytomas. A total of 24 tumors were treated and the median patient age was 52 years (range 29-70 years) at the time of initial CK therapy. The median follow-up period was 37 months (10-73 months) and all patients had been previously treated with surgical resection. Mean tumor volume was 9.16 cm^3 ^and the mean marginal and maximum radiosurgical doses to the tumors were 21.2 Gy and 26.8 Gy, respectively.

**Results:**

Of the 24 tumors treated, 22 have clinical follow-up data at this time. Of those 22 tumors, 12 decreased in size (54.5%), 6 remained unchanged (27.3%), and 4 showed recurrence (18.2%) after CK therapy. Progression-free survival rate was 95%, 71.5%, and 71.5% at 1, 3, and 5 years after multiple CK treatments. The 5-year survival rate after CK was 81%.

**Conclusions:**

CK is an effective and safe management option for hemangiopericytomas. The current series demonstrates a tumor control of 81.8%. Other institutions have demonstrated similar outcomes with stereotactic radiosurgery, with tumor control ranging from 46.4% to 100%.

## Background

Hemangiopericytomas (HPCs) are rare vascular tumors arising from Zimmerman pericytes associated with capillary walls. Central nervous systems HPCs are rare and account for 0.4% of primary CNS tumors and 2.4% of meningiomas[1,2]. Both clinically and radiographically, hemangiopericytomas resemble meningiomas but are known for their aggressiveness, high recurrence rates, and propensity for extracranial metastasis. Patients with HPCs present with a wide spectrum of symptoms, dependent upon location and histologic grade of the tumor.

Treatment of CNS HPCs is aggressive and consists of gross total resection combined with adjuvant radiotherapy[3]. Given the proposed cellular origin, dural sinus invasion, anatomic inaccessibility, and high vascularity of HPCs, gross total resection is often not sufficient. Maximal treatment consisting of gross total resection and radiotherapy conveys a mean survival of approximately 84 months from diagnosis[4].

Due to the potential for residual and recurrent tumor, stereotactic radiosurgery is well suited for post-operative adjuvant therapy, particularly for inaccessible locations[5]. The role of Gamma Knife (GKS) and CyberKnife (CK) in the treatment of hemangiopericytomas has been previously described with tumor control rates ranging from 46 - 100%[3,6-11]. Here within we report the Stanford University experience using CyberKnife stereotactic radiosurgery to treat fourteen patients with residual, metastatic, or recurrent CNS hemangiopericytomas.

## Materials and methods

### Patient Population

Fourteen patients were treated with CyberKnife stereotactic radiosurgery between the years 2002 and 2009 at Stanford University Medical Center. All patients were enrolled with approval from the Stanford Institutional Review Board (IRB) and in accordance with the Helsinki Declaration. Six patients were male (43%) and eight were female (57%) with a median age of 52 years (range 29 - 70 years) at the time of initial CK therapy (Table 1). All CNS HPCs were documented as residual, metastatic, or recurrent, post-resection lesions. Seven patients had undergone two or more surgical resections while seven patients had undergone only one prior operation. Nine patients had received prior cranial irradiation. Presenting symptoms correlated with lesion location and included headache, seizures, visual dysfunction, motor weakness and tandem gait. The mean time to CK treatment post-surgery was 7.6 years (range 1 month - 16 years).

**Table 1:** Summary of Patient Characteristics

**Ptn**.	Age at onset and gender	Clinical presentation	No. of surgery before CK	Radiation therapy before CK	Site	Grade	Time to CK post-surgery	No. of CK treatments	Follow-up (months)
1	43 M	HA, Vis	1	N	Torcular	3	2 yrs	1	73

2	39M	Vis	2	54Gy	Parasellar	-	16 yrs	2	36, 64

3	58M	Leg weak	2	45Gy	T6-8	3	6 yrs	1	37

4	47M	HA, Ataxia, Vis	1	N	Parafalcine	1	1 mo	1	37

5	42F	Leg weak, Sz	2	N	Parafalcine	-	10 yrs	1	39

6	29F	HA, Hand weak	1	N	Tentorium	1	1 mo	3	53, 30, 10

7	47F	Sen loss, Vis	1	GK	C- T- spine	-	10 yrs	1	26, 45

8	69F	Foot drop	1	N	Parafalcine	2	1 mo	1	41

9	38M	HA, Sz	1	50.4Gy	Left middle fossa	-	9 mo	1	59

10	51F	Left buttock pain	4	Y	Lumbar spine	-	16 yrs	3	15

11	41F	HA, Vis	2	59.4Gy	Rt Inf. Cerebellar	3	5 yrs	1	30

12	53F	Numbness, facial pain, diplopia	2	Y	Rt temporal, Cav Sinus	-	14 yrs	3	-

13	38F	Left facial palsy, tandem gait	2	54 Gy	Pineal space, Left Tentorium	-	15 yrs	2	-

14	35M	HA	1	Y	Posterior Fossa	-	12 yrs	2	15

(HA, headache; Vis, visual deficits; Sz, seizure; N, none; Y, prior radiation but no dosage available; GK, Gamma Knife)

### Tumor Characteristics

In total, the fourteen patients harbored twenty-four HPCs. Mean tumor volume was 9.16 cm^3 ^(range 0.03 - 56.7 cm^3^). Of the twenty-four total tumors treated, sixteen tumors required a single session treatment, four required two sessions, and four required three sessions or more. Tumors were located in a myriad of locations, including supra and infra-tentorial as well as spinal (Table 2).

**Table 2:** Summary of CyberKnife Radiosurgery Dosimetry

Ptn.	Age at CK	Tumor vol (cc)	Site	Marginal dose (Gy)	Isodose Line (%)	Fractions	Dmax (Gy)	% Target volume treated at/above dose	Conformality index	Tumor Control At last F/U
1	45	7.0	Torcular	30	75	1	25.32	97	1.38	R

2	52	3.62	Tentorium	22	80	3	27.5	96.5	1.33	D
	55	10.97	Petroclival	22	78	2	28.21	97	1.56	R

3	64	1.74	T6	24	78	3	30.77	96	1.47	R

4	47	3.5	Parafalcine	16	72	2	21.92	98	1.26	S

5	52	10.89	Parafalcine	20	73	2	27.4	97	1.46	S

6	29	1.5	Cav. Sinus	18		1	22.14			D
	33	1.12	Cav sinus	30	83	5	36.14	99	1.34	D
	37	0.97	Med. temp	20	76	1	26.3	99	1.21	R

7	57	0.23	C1	24	86	1	27.9		1.11	D
	59	0.16	C3-4	18	75	1				D
	60	0.03	T1	20	89	1	22.47	95	3.4	D
		0.07	T6	20	79	1	25.32	95	2.68	D
		0.06	T11	20	78	1	25.65	96	3.4	D

8	70	5.72	Parafalcine	22	79	1	27.85		1.6	D

9	39	21.8	Left middle fossa	16	77	1	21.92	97	1.1	S

10	67	39.8	L1	20	70	1	28.17	95	1.31	D
	67	0.99	L2	16	77	1	20.78	95	1.79	D
	67	8.52	L4	16	77	1	20.78	99	1.95	D

11	46	0.236	Rt. Inf Cerebellar	24	74	1	32.45	98.7	1.25	S

12	67	14.36	Rt. Mid Fossa	27	73	3	36.99	95.2	1.56	-

13	53	16.74	Pineal space, Left Tentorium	22	77	2	28.57	96.6	1.57	-

14	47	56.7	Posterior	21	76	1	27.63	95.1	1.19	S
		13.2	fossa	21	80	1	26.25	97.8	1.27	S

(R, recurrence; S, stable; D, decreased size)

### Treatment and Follow-up Evaluation

**Figure 1:**
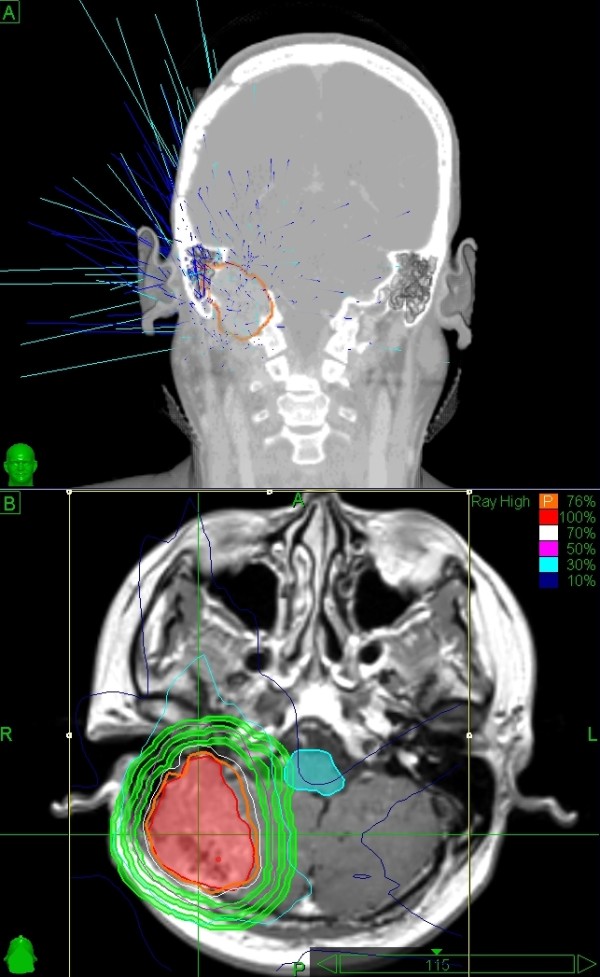
**CyberKnife contour for Patient 14, a forty-seven year old male who was treated for a 56.7 cm^3 ^in the posterior fossa**. A single fraction at marginal dose of 21 Gy and maximum dose of 27.6 Gy was used. The isodose line was 76% and the conformity index was1.19. At 15 months follow-up, the tumor was stable.

All fourteen patients and twenty-four tumors were treated with CyberKnife stereotactic radiosurgery (Accuray, Inc., Sunnyvale, CA). Patients were placed on the treatment bed and a previously designed facial thermoplastic mask was fitted for stabilization. Patients were then transferred to a CT scanner (Lightspeed; General Electric, Milwaukee, WI), where 125 ml of Omnipaque contrast was administered to obtain 1.25-mm slices of the lesion and its surrounding location. Patients then underwent a stereotactic MRI scan (2.0 mm slice thickness) with gadolinium contrast, which was then fused to the stereotactic CT scan. Tumor volume was carefully contoured and inverse treatment planning was performed to achieve a conformal treatment plan that minimized dose observed by adjacent eloquent structures (Figure 1).

For spinal treatments prior to 2005, patients first underwent implantation of either straight gold fiducials or stainless steel screws for tracking of spinal bony landmarks. Following implantation, the patient returned for a treatment planning CT. More recently, the development of the Xsight spine tracking system (Accuray Inc., Sunnyvale, CA) has eliminated the use of fiducial implantation. Instead, the system localizes spinal targets by direct reference to the adjacent vertebral structures.

During the actual treatment, the CyberKnife treatment algorithm places the LINAC at a determined position, using real-time imaging to locate the target and adjust for movements. The radiation beam is then delivered and the process repeated at various preset nodes surrounding the patient. Therefore, the target position is continually updated using x-ray image-to-image correlation, obviating the need for skeletal fixation to localize the target. The precision of localization is 0.3 mm, comparable with that which can be achieved by frame-based techniques.

Of the fourteen patients treated, follow-up data was available for twelve patients, accounting for a total of twenty-two tumors. Radiographic follow-up evaluation included gadolinium-enhanced MR images obtained every 4 months for the 1^st ^year after treatment, every 6 months during the 2^nd ^year, and annually thereafter. Clinical follow-up examination was conducted at the same intervals. The median clinical and radiographic follow-up period was 37 months (range 10-73 months).

## Results

### Imaging Outcome

The mean tumor volume was 9.16 cm^3 ^and the mean marginal and maximum radiosurgical doses to the tumors were 21.2 Gy (16 - 30 Gy) and 26.8 Gy (21.9 - 36.9 Gy), respectively. The mean isodose line was 77.5% (Table 3). Treatment regimens vary based on size of treated tumor, location to critical structures, and history of prior radiation. In this series, all patients with brain hemangiopericytomas who were treated with more than one session had their hemangiopericytoma located next to the brainstem, cavernous sinus, or optic pathways. A single lumbar spine hemangiopericytoma was treated in three sessions due to tumor size. Out of the twenty-four tumors treated, twenty-two have clinical follow-up data at this time. Of those twenty-two tumors, follow-up MRI showed twelve decreased in size (54.5%), six remained unchanged (27.3%), and four recurred or increased in size (18.2%) after CK therapy. Total tumor control rate was 81.8%. There does not appear to be a correlation between treatment dose, tumor volume, and tumor response in these patients. There was no radiological evidence of edema or necrosis in the tissue adjacent to the tumor in any patient in this series.

**Table 3:** Summary of Patient Characteristics and CyberKnife Dosimetry

Number of Patients	14
Male	6 (43%)

Female	8 (57%)

Number of Tumors	24

Number of Tumors with Follow-Up	22

Median Age	52 years (29 - 70 years)

Median Follow-Up	37 months (10 - 73 months)

Mean Tumor Volume	9.16 cm^3 ^(0.03 - 56.7 cm^3^)

Mean Marginal Dose	21.2 Gy (16 - 30 Gy)

Mean Maximum Dose	26.8 Gy (21.9 - 36.9 Gy)

Mean Isodose Line	77.5% (72 - 89%)

Mean Time to CK Post Surgery	7.6 years (1 month - 16 years)

Tumor Reduction	12 (54.5%)

Tumor Stable	6 (27.3%)

Tumor Recurrence	4 (18.2%)

Total Tumor Control	18 (81.8%)

### Clinical Outcome

Clinical symptoms were followed in all twelve patients. Of those with adequate follow up data, one patient reported resolution of headaches, eleven indicated no change in symptoms and zero patients described worsening of initial clinical presentation. All patients presenting with cranial nerve deficits remained as such with no improvement or worsening.

The patient with initial decrease (36 months follow-up) and subsequent increase in tumor size (64 months) had findings consistent with an ischemic event which left him with cognitive changes. The patient with documented tumor progression despite radiosurgery has since undergone three additional open surgeries to excise the anaplastic hemangiopericytoma. Unfortunately, his operations were complicated by hemorrhage and postoperative CSF leak, and his recurrent disease continues to cause visual decline and fatigue. Overall, the progression-free survival rate was 95%, 71.5%, and 71.5% at 1, 3, and 5 years after multiple CK treatments. The 5-year survival rate after CK treatment was 81%.

## Discussion

CNS hemangiopericytomas are malignant CNS lesions that exhibit aggressive behavior and are associated with high rates of local recurrence and distant metastasis. Surgical resection is the initial treatment of choice and carries an operative mortality of 9-24%[12,13].

In a recent systematic review of published literature by Rutkowski et al., several important prognostic factors influencing hemangiopericytoma mortality rates were identified[14]. Among the 563 patients reviewed, the overall median survival was 13 years, with 1-, 5-, 10-, and 20-year survival rates of 95%, 82%, 60%, and 23%, respectively. Gross total resection alone was associated with a median survival of 13 years, whereas subtotal resection resulted in a median survival of 9.75 years. Interestingly, in this report, postoperative adjuvant radiation was not associated with a superior survival benefit. Patients receiving >50 Gy of radiation had worse survival outcomes. Patients with tumors of the posterior fossa had a median survival of 10.75 vs. 15.6 years for those with tumors located elsewhere.

The primary challenge with surgical resection alone is the high rate of postoperative recurrence. Studies have shown a median rate of approximately 12 months. Although multiple resections are feasible, the appreciable morbidity associated with each intervention makes this option unattractive. Stereotactic radiosurgery combines the efficacy of resection with the more minimal rate of radiotherapy-induced morbidity. Some authors have asserted that the highly vascular nature of these tumors likely increases their favorable response to treatment[15]. The steep dose gradient achieved with stereotactic radiosurgery minimizes unintended radiation to eloquent structures[6].

### External Beam Radiotherapy Outcomes

External-beam radiotherapy has been used as adjuvant therapy for the treatment of local recurrences, often following surgical resection. At a focal fractionated dose of 50 Gy, studies have shown a significant increase in the length of time to tumor recurrence[1,7]. Dufour and colleagues demonstrated that postoperative external beam radiotherapy decreased the local recurrence rate to 12.5% compared to 88% after surgery alone[7]. Guthrie et al. reported that radiation therapy after surgical resection extended the mean time to recurrence from 34 to 75 months and extended survival from 62 to 92 months[1]. Glaholm and colleagues noted that even in those patients who had undergone resection previously, megavoltage photon irradiation alone improved neurological performance in 38% of patients, based on the Karnofsy performance score[16]. The authors of subsequent reports have also documented the benefit of radiotherapy in those previously treated with surgery, even when a gross-total resection had been achieved. Most recently, Shiariti and colleagues reported on 39 patients who underwent microsurgical resection with a mean follow-up period of 123 months[17]. External-beam radiation therapy extended the disease-free interval from 154 months to 254 months but was not effective in preventing metastasis. In those patients with EBRT and complete resection, the mean recurrence-free interval was found to be 126.3 months longer and overall survival 126 months longer than without EBRT.

### Stereotactic Radiosurgery

Eleven published studies (including this current series) on the use of stereotactic radiosurgery for recurrent and residual hemangiopericytomas have been reviewed in Table 4. Between the years of 1987 and 2010, a total of 137 patients with 241 lesions were treated with stereotactic radiosurgery and reported in the literature. For these lesions, the mean prescription dose was 16.2 Gy to the tumor margin, the mean follow-up period of 37.2 months, and the mean tumor control rate of 81.3%[3,6,8-11,15,16,18-20]. Since hemangiopericytomas are rare tumors, and many of them are treated with conventional radiation, our series size (fourteen patients with twenty-two tumors) is reasonable. As compared with several prior studies summarized in Table 4, it is notable that our study contributes to previous CyberKnife series on this rare tumor.

**Table 4:** Published Studies on Stereotactic Radiosurgery for Hemangiopericytoma

Series	Institution	Study period	Treatment Modality	No. of Patients/Lesions	Mean Marginal dose (Gy)	Mean Follow up (months)	Tumor control at last FU (%)
Coffey 1993[15]	Mayo Clinic	1990-1992	Gamma Knife	5/11	15.5	14.8	81.8

Galanis 1998[18]	Mayo Clinic	1976-1996	Gamma Knife	10/20	12-18	6-36	100*

Payne 2000[10]	U of Virginia	1991-1999	Gamma Knife	10/12	14	24.8	75

Sheehan 2002[3]	U of Pittsburgh	1987-2001	Gamma Knife	14/15	15	31.3	80

Chang 2003[6]	Stanford	1992-2002	LINAC, CyberKnife	8/8	20.5	44	75

Ecker 2003[8]	Mayo Clinic	1980-2000	Gamma Knife	15/45	16	45.6	93^

Kano 2008[20]	U of Pittsburgh	1989-2006	Gamma Knife	20/29	15	37.9	72.4

Sun 2009[11]	Beijing Neu. Ins.	1994-2006	Gamma Knife	22/58	13.5	26	89.7

Iwai 2009[19]	Osaka City Hosp	1994-2003	Gamma Knife	8/13	15.1	61	100

Olson 2010[9]	U of Virginia	1989-2008	Gamma Knife	21/28	17	69	46.4

**Veeravagu 2010**	**Stanford**	**2002-2009**	CyberKnife	**14/22**	**21.2**	**37**	**81.8**

*Tumors responded to GKS with decrease or stability in volume, but effect lasted less than 1 year in majority of patients. Study also includes the five patients from Coffey et al. 1993 manuscript.

^Also includes five patients from Coffey et al. 1993 manuscript.

In 1993, Coffey and colleagues from the Mayo Clinic provided the first preliminary SRS report for the treatment of hemangiopericytomas[15]. Five patients with eleven tumors were treated with GKS. At a mean marginal dose of 15.5 Gy and a short mean follow-up period of 14.8 months, the authors reported a tumor control rate of 81.8%. Galanis and colleagues added five more patients to the Coffey series for a total of 20 hemangiopericytomas[18]. Seven of the ten patients had previously undergone radiotherapy (dose range 3060-6400 cGy, median 5580 cGy) and all ten had undergone at least one prior surgical resection. Fourteen of the hemangiopericytomas decreased in size, four disappeared radiographically, and two were stable in size.

Payne et al. reported on ten patients with twelve lesions who had undergone treatment with GKS[10]. Nine of the patients had undergone prior craniotomies (mean number of surgeries 2.9) and four patients had undergone prior fractionated radiotherapy. With a mean peripheral dose of 14 Gy and mean follow-up period of 24.8 months, the authors demonstrated a 75% tumor control rate. Four of the nine tumors that decreased in size, however, subsequently increased in size after a mean of 22 months post-radiosurgery.

Sheehan et al. published a series on fourteen patients with fifteen hemangiopericytomas treated with GKS[3]. Twenty-seven prior surgical resections had been conducted in this population; seven patients had previously undergone radiotherapy. The marginal radiosurgery doses ranged from 11 to 20 Gy and the mean follow-up period was 31.3 months. At last follow-up, tumor regression was demonstrated in 80% of the fifteen tumors. Despite the effective local control rate, 29% of the patients developed remote lesions, indicating that radiosurgery provided little protection from metastatic spread. Similarly, other studies have indicated that metastatic disease is diagnosed between 63-99 months after the initial diagnosis[7,18]. The incidence of distant metastasis increases with time and has been reported as 13, 33, and 64% at 5, 10, and 15 years respectively[1].

Ecker and colleagues reported on fifteen patients with forty-five lesions who were treated with GKS[8]. Fourteen of these patients had previously undergone radiosurgery. At a mean marginal dose of 16 Gy, 93% of tumors had regressed or remained stable at the last follow-up. In total, nine patients eventually died due to metastatic disease and five patients died from tumor burden. Kano et al. published a series consisting of twenty patients who had undergone GKS for twenty-nine tumors[20]. A tumor control rate of 72.4% was reported at a mean follow-up period of 37.9 months. The mean marginal dose to the tumor periphery was 15 Gy. The authors reported that twelve patients (60%) were still alive at last follow-up while eight (40%) had died at average of 62.6 months following GKS therapy.

In a study by Sun and colleagues, twenty-two patients with fifty-eight foci underwent GKS at a mean tumor margin dose of 13.5 Gy[11]. Radiological follow-up at 26 months showed that 25 foci (43.1%) nearly disappeared, 13 foci (22.4%) reduced in size, 14 foci (24.1%) remained stable and 6 foci (10.3%) enlarged. The overall tumor control rate was 89.7%. Intracranial metastases developed in 7 patients (31.8%) and extracranial metastases developed in 3 patients (13.6%). Similarly, a much smaller study by Iwai et al. in 2009 demonstrated 66.7% tumor control at 34 months follow-up and a mean marginal dose of 13.7 Gy[19].

Recently, Olson and colleagues identified twenty-one patients with twenty-eight lesions who were treated with GKS[9]. These patients had received a mean marginal dose of 17 Gy and at last follow-up, the tumor control rate was 46.4%. The mean long term follow-up time of 69 months is greater than those of previous series assessing the role of radiosurgery in the treatment of hemangiopericytomas.

### The Stanford Experience

Chang and Sakamoto's series in 2003 confirmed those of earlier reports, demonstrating tumor control in 75% of the hemangiopericytomas treated during a mean 44 month follow-up period[6]. In this series, a LINAC based radiosurgery system was used to treat four tumors and CyberKnife radiosurgery was used to treat four tumors in a total of eight patients. The mean dose rates to tumor periphery in this series were slightly higher (20.5 Gy) compared with those in other series (16.2 Gy). The higher prescription dose, however, did not translate to increased tumor control rates or radiosurgery related complications.

The present series used CyberKnife to treat twenty-four tumors. A tumor control rate of 81.8% was achieved with a mean follow-up of 37 months. Although the mean marginal dose is 21.2 Gy (the highest amongst published series), adverse effects of radiotherapy were not observed. Progression-free survival rate was 95%, 71.5%, and 71.5% at 1, 3, and 5 years after multiple CK treatments. The 5-year survival rate after stereotactic radiosurgery was 81%. As is the case in other series, all patients had previously undergone either single or multiple craniotomies for attempted gross total resection.

Conclusions from the Stanford study are similar to those made by other groups. Stereotactic radiosurgery is a focal, localized treatment modality and does not prevent metastases, intracranial or otherwise. Metastases outside the treatment area often developed within a few years after initial treatment, but in one case was reported to appear after twenty-two years[12]. Due to the aggressive nature of hemangiopericytomas, initial decreases in tumor size or even disappearance can be followed by re-growth. This was observed in the present study and also noted previously[10]. Both of these issues support the need for close clinical and radiographic follow-up in this patient population.

## Conclusion

Hemangiopericytomas are known for their aggressive pathology, high recurrence rate, and propensity for distant metastasis. Surgical resection remains the initial treatment option; however, postoperative stereotactic radiosurgery has been shown to be effective in increasing time to recurrence as well as patient survival. As suggested by this series and previous reports, stereotactic radiosurgery, including CyberKnife radiosurgery, results in effective tumor control (tumor control rates ranging from 46.4% to 100%, Stanford 81.8%). Close clinical and radiographic follow-up is necessary due to the high probability of local recurrence and distant metastases. Because radiosurgery is a focal treatment, it does not eliminate the possibility of regional or distant metastases, which remain sources of significant morbidity and mortality for these patients.

## Competing interests

The authors declare that they have no competing interests.

## Authors' contributions

AV and BJ carried out the data analysis, literature review, and manuscript drafting. CP provided critical revisions of the manuscript. ML, SS, IG, and SC participated in data collection, data analysis, and enrolling clinical cohorts. SC conceived of the study and participated in its design and coordination and helped to draft and review the manuscript. All authors read and approved the final manuscript.
